# ^23^Na chemical shift imaging and Gd enhancement of myocardial edema

**DOI:** 10.1007/s10554-012-0093-6

**Published:** 2012-07-12

**Authors:** Eissa N. E. Aguor, Cees W. A. van de Kolk, Fatih Arslan, Marcel G. J. Nederhoff, Pieter A. F. M. Doevendans, Gerard Pasterkamp, Gustav J. Strijkers, Cees J. A. van Echteld

**Affiliations:** 1 Laboratory of Experimental Cardiology, University Medical Center Utrecht (UMCU), Utrecht, The Netherlands; 2 ICIN-Netherlands Heart Institute, Utrecht, The Netherlands; 3 Department of Cardiology, University Medical Center Utrecht (UMCU), Utrecht, The Netherlands; 4 Biomedical NMR, Department of Biomedical Engineering, Eindhoven University of Technology, Eindhoven, The Netherlands; 5 Hafenrainstrasse 103, 4104 Oberwil, Switzerland

**Keywords:** Myocardial edema, Hypertension, ^23^Na CSI, CE MRI, Endocardial enhancement

## Abstract

Myocardial edema can arise in several disease states. MRI contrast agent can accumulate in edematous tissue, which complicates differential diagnosis with contrast-enhanced (CE)-MRI and might lead to overestimation of infarct size. Sodium Chemical Shift Imaging (^23^Na-CSI) may provide an alternative for edema imaging. We have developed a non-infarct, isolated rat heart model with two levels of edema, which was studied with ^23^Na-CSI and CE-MRI. In edematous, but viable tissue the extracellular sodium (Na_e_^+^) signal is hypothesized to increase, but not the intracellular sodium (Na_i_^+^) signal. Isolated hearts were perfused at 60 (n = 6) and 140 mmHg (n = 5). Dimethyl methylphosphonate (DMMP) and phenylphosphonate (PPA) were used to follow edema formation by ^31^P-MR Spectroscopy. In separate groups, Thulium(III)1,4,7,10 tetraazacyclododecane-*N*,*N*′,*N*″,*N*′′′-tetra(methylenephosphonate) (TmDOTP^5−^) and Gadovist were used for ^23^Na-CSI (n = 8) and CE-MRI (n = 6), respectively. PPA normalized signal intensity (SI) was higher at 140 versus 60 mmHg, with a ratio of 1.27 ± 0.12 (*p* < 0.05). The (DMMP-PPA)/dry weight ratio, as a marker of intracellular volume, remained unchanged. The mid-heart cross sectional area (CSA) of the left ventricle (LV) was significantly increased at 140 mmHg. In addition, at 140 mmHg, the LV Na_e_^+^ SI increased with a 140 mmHg/60 mmHg ratio of 1.24 ± 0.18 (*p* < 0.05). Na_i_^+^ SI remained essentially unchanged. With CE-MRI, a subendocardially enhanced CSA was identified, increasing from 0.20 ± 0.02 cm^2^ at 60 mmHg to 0.31 ± 0.02 cm^2^ at 140 mmHg (*p* < 0.05). Edema shows up in both CE-MRI and Na_e_^+^. High perfusion pressure causes more edema subendocardially than subepicardially. ^23^Na-CSI is an attractive alternative for imaging of edema and is a promising tool to discriminate between edema, acute and chronic MI.

## Introduction

Myocardial interstitial edema can arise in several disease states or following clinical interventions, including myocardial ischemia and infarction [1–11], arterial [12] and pulmonary hypertension [13], myocarditis [14], cardio-pulmonary bypass and cardioplegic arrest [15] and cardiac transplantation [16]. The causes of edema may vary: in patients with hypertension, differences in hydrostatic pressure may dominate, whereas in patients with coronary artery disease, differences in osmotic pressure and microvascular permeability are probably more important [17]. ^1^H-based MRI methods such as contrast-enhanced (CE)-MRI and T_2_-weighted MRI are valuable tools to assess injured myocardium. Indeed, CE-MRI has become the gold standard to delineate chronic myocardial infarction [18, 19]. However, the accuracy of CE-MRI to delineate acute myocardial infarction has been questioned because of the accumulation of contrast agent in the expanded extracellular compartment of both infarct and peri-infarct regions [1–4, 14]. Apparently, in this phase CE-MRI much resembles T_2_-weighted MRI, which has shown great promise to delineate the area at risk (AAR) and to quantify myocardial salvage in patients with acute coronary syndromes after events related to the formation of edema [5–11]. However, both these methods depend on relative regional differences in myocardial signal intensity and are perhaps less suitable to identify globally edematous tissue associated with any of the other disease states mentioned above, such as hypertension or myocarditis.

An alternative approach to assess myocardial edema is imaging of myocardial sodium with ^23^Na-MRI, since we expect the extracellular sodium (Na_e_^+^) signal to increase with increasing interstitial edema because of the expansion of the extracellular space. In a number of studies an elevated total sodium (Na_t_^+^) signal has been observed after myocardial infarction, both in humans and animals [19–22] Particularly, imaging intra- (Na_i_^+^) and extra- (Na_e_^+^) cellular sodium separately can provide more precise information to assess the sodium gradient and cell membrane integrity [23]. This can be achieved with chemical shift imaging (^23^Na-CSI) in combination with a paramagnetic chemical shift agent to separate Na_i_^+^ from Na_e_^+^ signals [23, 24].

We hypothesize that, in viable tissue with cell membranes still intact but with a high level of extracellular edema, Na_i_^+^ signal intensity is normal because viable cells should be able to maintain a normal Na^+^ gradient and a normal intracellular volume, whereas Na_e_^+^ and CE-MRI signal intensities will be increased because of the larger extracellular space. To characterize edema independent from ischemia, this study consisted of two parts. First, using phosphorus-31 magnetic resonance spectroscopy (^31^P MRS), we developed an isolated heart model with two levels of extracellular edema based on crystalloid perfusion at different pressures. Secondly, we aimed to characterize edema in non-ischemic myocardial tissue with CE-MRI and ^23^Na-CSI.

## Methods

### Animal and heart preparation

Male Wistar rats (n = 25, body weight = 300–350 g) were anesthetized with isoflurane inhalation and given heparin (500 IU/Kg i.v.). Subsequently, the hearts were rapidly excised and placed in modified, ice-cold Krebs-Henseleit (KH) buffer. After cannulation of the aorta, perfusion was initiated according to Langendorff at a constant pressure (60 mmHg) with KH at 37 °C. To further prepare the heart, a drain was inserted through the apex via the mitral valve to remove Thebesian flow. A latex balloon was inserted into the left ventricle through the mitral valve and filled with water to reach a left ventricular end-diastolic pressure (LVEDP) of 7.5 mmHg. Two pressure transducers (MP-15, Micron Instruments) were used to monitor perfusion pressure and to measure the isovolumic contractile pressure of the left ventricle via the inserted balloon. Left ventricular developed pressure (LVPD) was determined as the difference between left ventricular systolic pressure (LVSP) and LVEDP. To maintain a heart rate of 300 beats/min, the outflow tract of the right ventricle was connected with two copper-wire electrodes to a (4–7 V, 0.5 ms) stimulator (model S88, Grass Instruments, Quincey, MA, USA). Heart rate was determined from the pressure curve. Coronary flow was measured with a flow probe (Skalar instruments, Delft, The Netherlands). The data were recorded with a PowerLab Data Acquisition System (ADI Instruments, Australia). Hearts were placed in a 20-mm-diameter NMR tube and positioned inside the MR scanner. The experimental protocol was in accordance with the guidelines of the Committee for Animal Experiments of the University Medical Center Utrecht, The Netherlands.

### Perfusion solutions

The modified KH buffer contained (in mmol/L): 119.0 NaCl, 4.7 KCl, 1.0 MgCl_2_, 24.0 NaHCO_3_, 1.3 CaCl_2_, 11.0 glucose, and 5.0 Na-pyruvate. Before perfusion, the KH buffer was filtered through 8 μm (Millipore, Bedford, MA) filters. The solution was equilibrated with 95 % O_2_ and 5 % CO_2_, resulting in a pH of 7.4 at 37 °C, prior to the addition of the CaCl_2_. For ^31^P-MRS, we included in the buffer 10 mM dimethyl methylphosphonate (DMMP), which distributes evenly in intra- and extracellular spaces, as a marker of total water space and 10 mM phenylphosphonate (PPA), which is non-permeant, as an extracellular space marker [25]. In the ^31^P spectra, the DMMP and PPA signals can be easily discriminated from endogenous phosphate signals. The difference between DMMP and PPA signals was calculated to determine the intracellular space. After preparations, hearts were allowed to stabilize at 60 mmHg. For ^23^Na-CSI, Thulium(III)1,4,7,10 tetraazacyclododecane-*N*,*N*′,*N*″,*N*′′′-tetra(methylenephosphonate) (TmDOTP^5−^, 3.5 mM) [26] was included in the buffer as a shift reagent (SR), to separate intra and extra-cellular Na^+^ signals. TmDOTP^5−^ is also non-permeant and therefore only interacts with Na_e_^+^, causing a shift in resonance frequency and leaving the Na_i_^+^ resonance at its original frequency.

To correct for Ca^2+^ binding by the shift reagent, the total Ca^2+^ added to the buffer was increased to 3.42 mM, which resulted in a free [Ca^2+^] of 0.85 mmol/L as measured by a HI 4004-51 Ca^2+^ Ion Selective Electrode (ISE) connected to a pH/ISE meter (HI 3221, Hanna instruments). For CE-MRI scans, 1.3 mM Gadobutrol (Gadovist, Bayer Schering Pharma AG, Berlin, Germany) was included in the buffer.

### Modification of Langendorff set up

Usually, we aspirate the effluent from a level above the heart to keep the hearts submerged for better magnetic susceptibility matching. However, the effluent generates unwanted extra-cardiac ^23^Na and ^31^P signals. Two approaches were used to reduce these unwanted signals. For the ^31^P-MRS study, the effluent was removed from the bottom of the NMR tube, sacrificing the susceptibility matching. For the ^23^Na-CSI study, an additional perfusion line ending at the bottom of the tube was used to flush the tube at a rate of 30 ml/min with a buffer in which Na^+^ was replaced by Li^+^. In this case, the hearts were kept submerged.

### MRS and MRI protocols

**Fig. 1:**
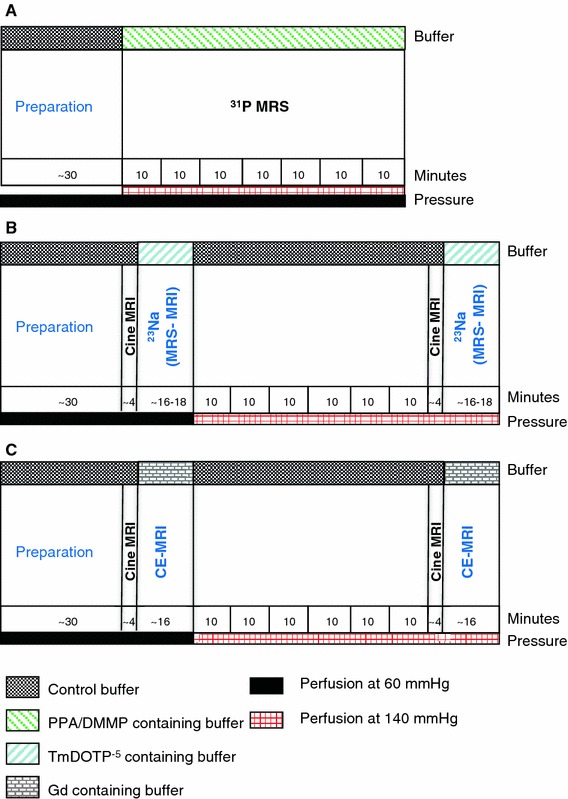
Schematic representation of experimental protocols. **a** ^31^P-MRS, **b** ^23^Na-chemical shift imaging (^23^Na-CSI), **c** contrast-enhanced MRI (CE-MRI)

A vertical 9.4 T, 89 mm diameter bore scanner was used, equipped with a 1,500 mT/m gradient system, which was interfaced with an AVANCE 400 DRX spectrometer (Bruker, Germany). All heart preparation including coil tuning and matching and optimization of magnetic field homogeneity was performed at a perfusion pressure of 60 mmHg and took 30–34 min as indicated in Fig. 1.

#### ^31^P-MRS

Phosphorus spectroscopy was performed with a 20 mm multi-nuclear probehead (Bruker, Germany). ^31^P spectra were acquired for 10 min by accumulation of 32 consecutive free induction decays (FIDs) following 90° pulses, using 2,048 data points and 12.9 kHz spectral width (SW). Pulse repetition time was 18.75 s to achieve a fully T_1_ relaxed signal.

#### CE-MRI and ^23^Na-CSI

The imaging protocol consisted of the following steps. First, 2- and 4-chamber view scout scans were obtained with a ^1^H 20-mm-diameter birdcage coil (Bruker, Germany) to plan a single mid-ventricular short-axis slice. Next, cine imaging was performed with a self-gated gradient-echo sequence (TE = 1.9 ms, TR = 5.2 ms, number of movie frames = 15, slice thickness = 2.5 mm, matrix = 256 × 256, field of view (FOV) = 2 × 2 cm^2^). Gated CE-MRI scans were performed with T_1_-weighted short-axis multi-slice FLASH sequence, with the following parameters: TR = 63 ms, TE = 2.8 ms, FA = 75°, NA = 8, 6 slices of 2.5 mm, matrix = 256 × 256, FOV = 2 × 2 cm^2^. To monitor the arrival of Gd containing buffer, eight serial CE-MRI scans were performed. The last scan in this series was used for edema analysis.

For ^23^Na-CSI, the ^1^H birdcage coil was replaced with a ^23^Na 20-mm-diameter birdcage coil (Bruker, Germany). To verify the heart position, scout images were obtained and subsequently a 5 mm mid-axial ventricular slice was acquired as a reference image with a ^23^Na self-gated FLASH sequence (TE = 2.4 ms, TR = 25 ms and 128 repetitions, 50° FA and SW = 7,714 Hz, matrix = 32 × 32 and FOV = 2 × 2 cm^2^). To monitor the shift of Na_e_^+^ after switching to the TmDOTP containing buffer, ^23^Na MR spectra were acquired with a 75° pulse, SW = 5.5 kHz, TR = 105 ms, and 512 data points. Subsequently, acquisition weighted 2D-^23^Na CSI was performed with the following parameters: 10,000 acquisitions, number of averages (NA) in center of k-space = 55, TR = 30 ms, 90° 0.5 ms sinc pulse, BW = 12,420 Hz, FOV = 2 × 2 cm^2^, matrix = 16 × 16 and reconstruction matrix = 32 × 32. FIDs were collected with 128 complex data points and 100.8 μs dwell time resulting in an acquisition time of 25.8 ms/FID. Gradient switching resulted in an acquisition delay of 0.91 ms.

### Experimental protocol

Two studies were performed. First, we used ^31^P-MRS to monitor the formation of edema in 2 groups of isolated hearts. Secondly, edema was characterized with CE-MRI and ^23^Na-CSI. Schematic diagrams of the experimental protocols for the 2 studies are shown in Fig. 1.

#### ^31^P-MRS group

After preparing the heart at 60 mmHg perfusion pressure, hearts were continuously perfused with PPA/DMMP-containing buffer for 70 min (Fig. 1a) either at 60 mmHg, n = 6 or at 140 mmHg, n = 5, to induce different levels of edema. As depicted in Fig. 1a, ^31^P MR spectra were acquired as an average over 10 min to track the change of PPA and DMPP signals and to measure the extent of extra-cellular edema during these 70 min of perfusion.

#### ^23^Na-CSI group

Hearts (n = 8) were perfused at 60 mmHg and subsequently at 140 mmHg for 60 min (Fig. 1b). After stabilization of the isolated hearts at 60 mmHg, ^1^H scout images were acquired followed by 2- and 4-chamber cine MRI. Thereafter, a cine scan was acquired for a mid-axial slice. Subsequently, the hearts were perfused with buffer containing SR and ^23^Na spectra were acquired to monitor the arrival of buffer containing SR. ^23^Na scout images were obtained to verify the anatomical position of the heart. Then a reference image of a mid-axial slice was acquired, followed by CSI of the same slice. The same scan protocol was repeated after 60 min of perfusion at 140 mmHg.

#### CE-MRI group

Hearts (n = 6) were perfused at 60 mmHg (Fig. 1c) and subsequently at 140 mmHg for 60 min. After stabilization of the isolated hearts at 60 mmHg, ^1^H scout images were acquired, followed by 2-chamber and 4-chamber cine MRI. Next, a cine scan was acquired for a mid axial slice. Subsequently, the hearts were perfused with buffer containing Gadovist. Eight serial CE-MR scans were acquired every 2 min at 60 mmHg to monitor the arrival of Gadovist containing buffer. The same MR scan protocol was repeated after 60 min of perfusion at 140 mmHg.

### Data analysis

For ^31^P-MRS, all spectra were analyzed with jMRUI 4.0 software. After a polynomial baseline correction, the spectra were fitted with AMARES, a time-domain based fitting algorithm. The ^23^Na-CSI data were zero-filled in both the spatial and spectral domain resulting in a 256 × 64 × 64 reconstruction size. To optimize peak separation a Lorentzian-Gaussian multiplication was applied. Epi- and endocardial contours of the left ventricle were manually drawn on end-diastolic ^1^H images to calculate the left ventricular cross sectional area (CSA). Subsequently, these contours were copied to the ^23^Na-CSI images, to determine the Na_e_^+^ and Na_i_^+^ average signal intensities of the entire left ventricle. For CE-MRI, Epi- and endo-cardial LV contours were drawn on the ^1^H axial cine images and then copied to CE-MR images. Subsequently, the CSA of the hyperenhanced areas were delineated manually. The CSA and the signal intensities of both the entire LV myocardium and the hyperenhanced areas were determined with Bruker Paravision 4 software.

### Tissue water content

At the end of the MRI measurements, the hearts were cut open and blotted dry with a soft tissue. The hearts were then weighed to determine their wet weights. Subsequently, the dry weights of the hearts were measured after desiccation in an oven at 50 °C for 48 h. The wet/dry (W/D) weights of the isolated rat hearts were then determined.

### Statistical analysis

Results were expressed as mean ± standard error of the mean for each group of animals. A paired *t* test was performed to compare mean values for each time point post perfusion. Results were considered significantly different for a two-tailed p value of less than 0.05. The correlation test of PPA values against W/D ratios was performed using a Pearson correlation with two-tailed p values.

## Results

### ^31^P-MRS

The addition of PPA and DMMP to the perfusate and the non-submerged state of the heart did not affect the stability of the heart, in terms of steady heart rate and coronary flow. The “dry” setup reduced the shimming quality as expected. The coronary flow was 24.0 ± 0.5 ml/min for the 140 mmHg group and 9.9 ± 0.4 ml/min for the 60 mmHg group.

**Fig. 2:**
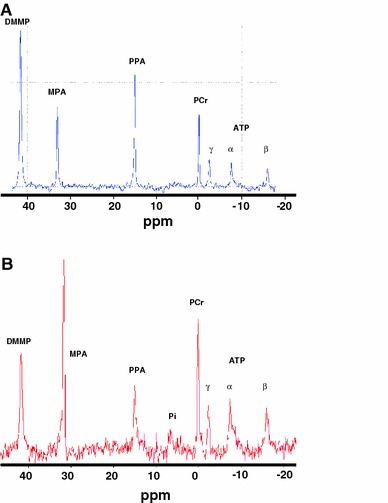
Typical examples of ^31^P-MRS spectra of an isolated rat heart, **a** while the heart is submerged in buffer and **b** after removing the effluent. The signal of the three phosphate groups of ATP, phosphocreatine (PCr), phenylphosphonate (PPA) as extracellular space marker, methylphosphonate (MPA), a solution in a small glass capillary as a reference, and dimethyl methylphosphonate (DMMP) as a marker of total water space are pointed out. MPA reference volume in (**b**) was different from that in (**a**)

**Fig. 3:**
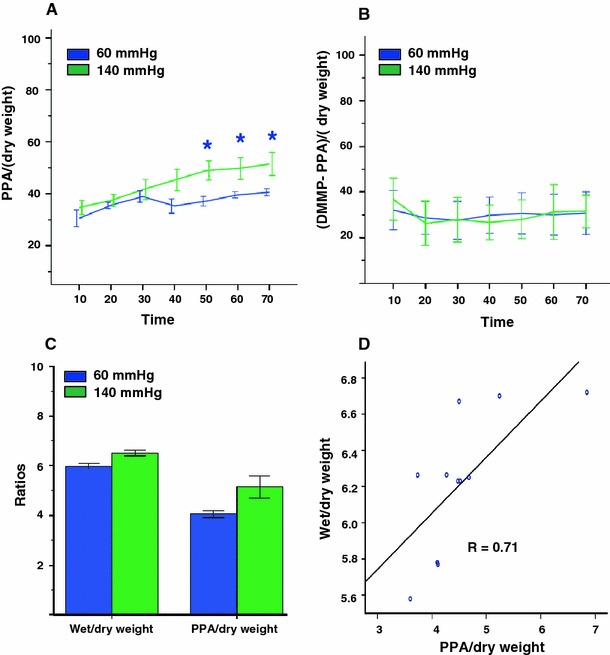
^31^P-MRS data on extra- and intracellular space markers and heart wet/dry weights from isolated rat hearts perfused at 60 and 140 mmHg. **a** PPA/heart dry weight as a function of time. **b** (DMMP-PPA)/heart dry weight as a function of time. **c** Normalized heart wet/dry and PPA/heart dry weight ratios 70 min after switching to the phosphonates containing buffer. **d** Heart wet/dry weight ratio versus PPA/heart dry weight ratio

Figure 2 shows examples of ^31^P MR spectra of an isolated rat heart (Fig. 2a) still submerged in the NMR tube and (Fig. 2b) after the effluent was removed from the bottom of the tube, both during perfusion with perfusate containing PPA and DMMP. Figure 3a summarizes the PPA signal intensities normalized by heart dry weight (PPA/dry weight), whereas Fig. 3b depicts the difference between DMMP and PPA signal intensities normalized by heart dry weight [(DMMP-PPA)/dry weight], both as function of time for the 60 and 140 mmHg groups. Ten minutes after switching to the phosphonates-containing buffer, PPA/dry weight was 30.6 ± 3.2 and 34.7 ± 2.7 for 60 and 140 mmHg, respectively. From 50 min onward, PPA/dry weight differences between the 60 and 140 mmHg group were significant (*p* < 0.05). After 70 min PPA/dry weight was 51.5 ± 4.5 at 140 mmHg versus 40.6 ± 1.4 at 60 mmHg, with a 140 mmHg/60 mmHg ratio of 1.27 ± 0.12. The calculated (DMMP-PPA)/dry weight, as a marker for the intracellular space, remained at approximately baseline values (29.9 ± 2.1) during the entire perfusion period for both groups.

The higher level of edema, deduced from a larger PPA signal at 140 mmHg, was corroborated by an increase in heart wet/dry weight ratio (W/D) of the 140 mmHg group in comparison to the 60 mmHg group (Fig. 3c, 6.5 ± 0.1 vs. 6.0 ± 0.1, respectively, *p* = 0.05). Moreover, a positive, significant correlation was found between W/D and PPA/dry weight (Fig. 3d, Pearson correlation coefficient 0.71, *p* = 0.02).

### ^23^Na-CSI

**Fig. 4:**
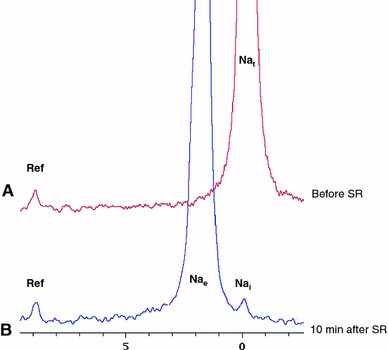
^23^Na-NMR spectra acquired with control buffer (**a**) and with shift reagent (SR) TmDOTP containing buffer. In the absence of TmDOTP (control buffer, **a**), the on-resonance was seen at 0 ppm. After 10 min of continuous perfusion with TmDOTP, Na_e_^+^ peak was separated from Na_i_^+^ peak. Reference: a small glass capillary, which contained a known amount of TmDOTP

Baseline cine MRI and ^23^Na-MRI were performed at 60 mmHg perfusion pressure, after which the perfusion pressure was increased to 140 mmHg (Fig. 1b). The hearts remained stable during perfusion with SR (TmDOTP). However, immediately after switching to SR-containing buffer, the hearts left ventricular developed pressure (LVDP) dropped from 60.0 ± 4.0 mmHg to 34.0 ± 4.3 (*p* < 0.05) at 60 mmHg and from 99.4 ± 6.5 to 48.0 ± 4.3 mmHg (*p* < 0.05) at 140 mmHg as a result of the reduction of the free Ca^2+^ concentration due to the affinity of the shift reagent to Ca^2+^ [26]. During perfusion with SR-containing buffer, two ^23^Na peaks were observed in the acquired spectra (Fig. 4). A large peak downshifted by 2.2 ± 0.2 ppm corresponding to Na_e_^+^ and mainly representing signal from the vasculature and interstitium, and the Na_i_^+^ intracellular sodium peak at 0 ppm. Similar Na_i_^+^ and Na_e_^+^ peaks were seen in the localized spectra of the CSI data.

**Fig. 5:**
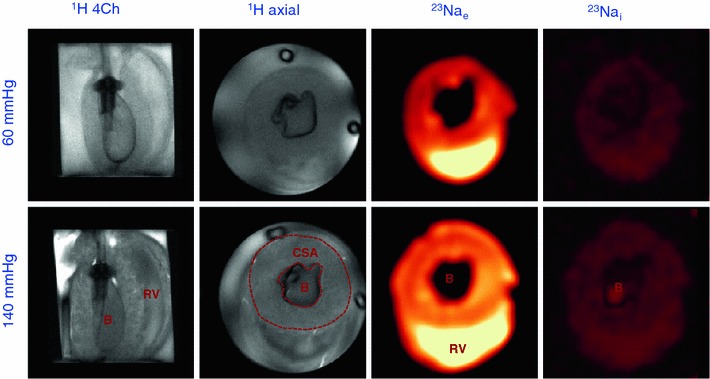
Representative MR images of an isolated rat heart perfused at 60 and 140 mmHg. Single frame ^1^H-cine MRI and extracellular and intracellular ^23^Na-CSI. *B* balloon, *CSA* cross sectional area of the LV, *RV* right ventricular lumen

Figure 5 shows ^1^H longitudinal and axial views of an isolated rat heart and corresponding Na_e_^+^ and Na_i_^+^ images at 60 and 140 mmHg. The balloon in the left ventricle was filled with Na^+^-free solution and therefore appeared dark in ^23^Na-CSI images. The right ventricular cavity had very high signal intensity, because it was filled with Na^+^-containing effluent. The Na_i_^+^ images were of considerable lower intensity than the Na_e_^+^ images because of the lower Na_i_^+^ concentrations in the myocardium. As can be readily seen in the cine ^1^H MR images (and in the ^23^Na images), the CSA of the mid-axial LV myocardium showed a considerable increase at 140 mmHg, going from 0.66 ± 0.03 cm^2^ at 60 mmHg to 0.91 ± 0.03 cm^2^ at 140 mmHg (*p* < 0.05) with a 140 mmHg/60 mmHg ratio of 1.40 ± 0.08, in good agreement with the PPA data.

In addition to the increase of the LV CSA, also an increase of LV Na_e_^+^ relative signal intensity was observed, going from 17.4 ± 1.7 at 60 mmHg to 21.6 ± 2.2 at 140 mmHg (*p* < 0.05), with a 140 mmHg/60 mmHg ratio of 1.24 ± 0.18 (*p* < 0.05), in good agreement with CSA and PPA data. We also found an increase in LV Na_i_^+^ relative signal intensity, going from 1.8 ± 0.2 at 60 mmHg to 2.0 ± 0.2 at 140 mmHg (*p* < 0.05), with a 140 mmHg/60 mmHg ratio of 1.11 ± 0.17. However, the shift reagent resulted in a clear but not complete separation between Na_e_^+^ and Na_i_^+^ CSI signals. The Na_e_^+^ signal is at least an order of magnitude larger than the Na_i_^+^ signal, and spectral deconvolution shows that the increase in the Na_i_^+^ signal can be explained for >80 % by the overlap with the increased Na_e_^+^ signal. Therefore, the corrected LV Na_i_^+^ signal shows no change when going from 60 to 140 mmHg.

### CE-MRI

**Fig. 6:**
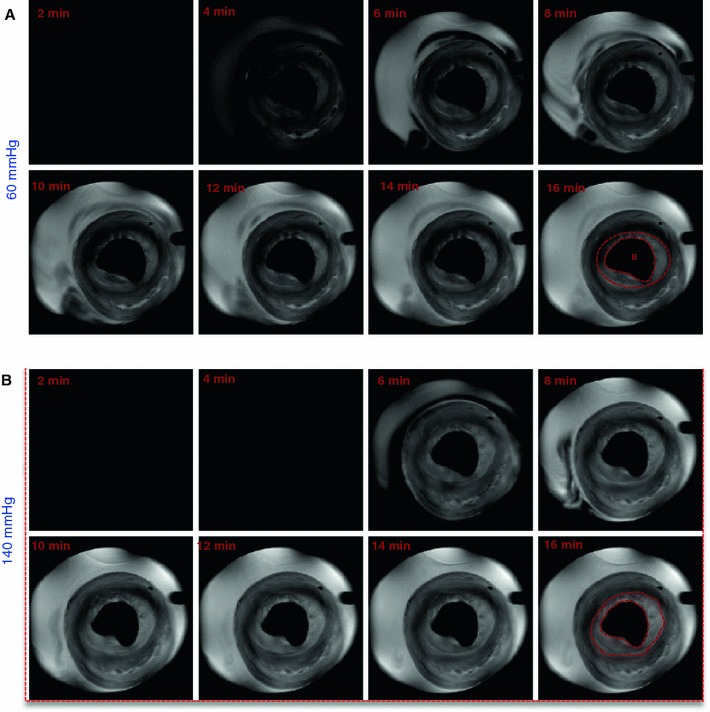
Representative T_1_-weighted MR images at 60 and 140 mmHg of a mid-axial slice of an isolated rat heart. Scans were acquired every 2 min after switching to Gadovist containing buffer. *B* balloon

After switching to the buffer with Gadovist, heart function remained stable. Baseline cine MRI and CE-MRI were performed at 60 mmHg, after which the perfusion pressure was increased to 140 mmHg. Figure 6 shows a series of T_1_ weighted MR images every 2 min after switching to Gd-containing buffer at 60 and 140 mmHg. The Gd started to appear in both groups after 4 min. In addition, CE-MR images showed an increased subendocardial enhanced area compared to the rest of the myocardium at 60 mmHg and even more so at 140 mmHg. From 10 min onwards the signal intensity of this hyper-enhanced area, delineated with a red dashed line, remained constant.

**Fig. 7:**
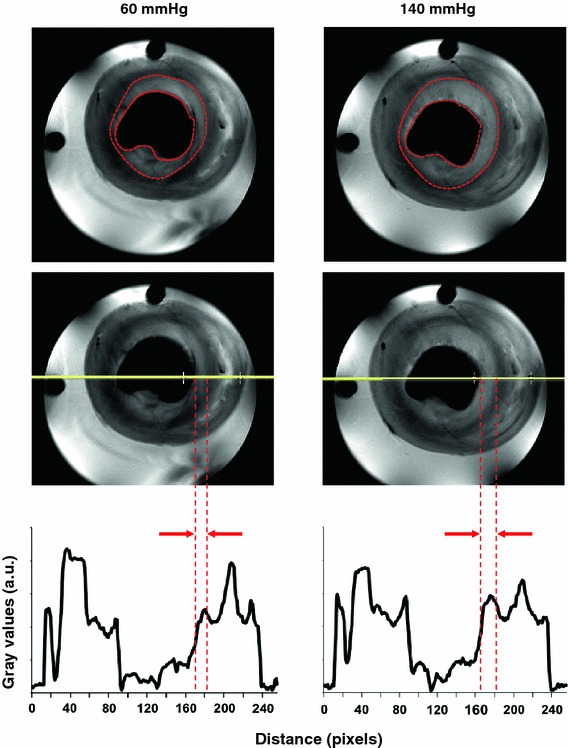
Representative CE-MR images for the same mid-axial slice of an isolated rat heart perfused **a** at 60 mmHg and **b** at 140 mmHg. **c** The signal profiles corresponding to the red lines across the images. The enhanced subendocardial area (*marked* with *red dashed line*) at 140 mmHg was larger than at 60 mmHg (*p* = 0.01), which can also be appreciated from the signal profile (**c**). The signal intensity of the subendocardial area was higher than the epicardial area both at 60 and 140 mmHg (*p* < 0.03)

Figure 7 shows typical CE-MR images at the beginning of the 60 mmHg perfusion and later at 140 mmHg and corresponding signal profiles across the images. As can be readily seen in the CE-MR image, the CSA of the hearts during perfusion at 140 mmHg was increased. From the cine images, the CSA of the mid-axial slice of the LV myocardium was significantly (p = 0.03) increased from 0.74 ± 0.04 cm^2^ at 60 mmHg to 0.90 ± 0.03 cm^2^ at 140 mmHg, with a 140 mmHg/60 mmHg ratio of 1.22 ± 0.08.

The enhanced subendocardial area (Fig. 7, region outlined by the red dashed line) at 140 mmHg was significantly larger than at 60 mmHg (0.20 ± 0.02 cm^2^ vs. 0.31 ± 0.02 cm^2^, *p* = 0.01), with a ratio of 1.55 ± 0.07. Relative signal intensities in these enhanced areas were 15.1 ± 0.7 at 60 mmHg and 16.3 ± 1.0 at 140 mmHg (*p* > 0.05), with a ratio of 1.08 ± 0.05. The relative signal intensities between the subendocardial area and the subepicardial area were significantly different at both 60 mmHg (subendocardial 15.1 ± 0.7 vs. subepicardial 13.4 ± 0.6, *p* < 0.03, ratio 1.13 ± 0.01) and 140 mmHg (subendocardial 16.3 ± 1.0 vs. 14.3 ± 0.9, *p* < 0.03, ratio 1.14 ± 0.01).

## Discussion

Using ^31^P-MRS we have developed a non-infarct model of extracellular edema in isolated rat hearts. With this model, we have demonstrated that CE-MRI can identify edematous myocardial tissue, most likely as a result of an increased distribution volume of Gd. Surprisingly, we found more edema in the subendocadial area than in the subepicardial area. We have demonstrated in this same edema model that the Na_e_^+^ signal increases with increasing edema, confirming that Na_e_^+^ is a useful endogenous marker of extracellular volume. Interestingly, a similar transmural distribution of signal intensities as observed in the contrast-enhanced images was also observed in the Na_e_^+^ images. Na_i_^+^ essentially remained unchanged in edema.

Using ^31^P-MRS we have shown that perfusion of hearts for an hour with crystalline buffers at high perfusion pressures leads to formation of interstitial edema, as evidenced by an increased distribution volume for PPA (Fig. 3). In addition, we observed a significant correlation between the increased PPA signal and the heart wet/dry weight ratio at the end of experiment. The increased PPA signal and the heart wet/dry ratio in our experiment are in line with findings by Li et al. [27]. The intracellular space remained unchanged, as evidenced by the stability of the difference between DMMP and PPA signals. This is in contrast to the results of Li et al., who observed intracellular edema as well, but in addition to simultaneous arterial/venous perfusion, they have used a hyperkalemic buffer, which may have contributed to the intracellular edema.

The presence of edema could also be confirmed by cine ^1^H-MRI. The CSA in both ^23^Na-CSI (Fig. 5) and CE-MRI (Fig. 7) groups showed a significant increase at 140 mmHg compared to 60 mmHg perfusion pressure, indicating an expanded volume of the extracellular compartment caused by myocardial interstitial edema. The expanded extracellular compartment is in line with the increased PPA signal and the heart wet/dry weight ratio of the ^31^P-MRS study.

In parallel to the increased CSA, the LV Na_e_^+^ signal intensity increased at 140 mmHg, corroborating an increased level of interstitial edema. The absence of intracellular edema in the used model, as found with the ^31^P-MRS intracellular space measurements, was corroborated by the essentially unchanged LV Na_i_^+^ signal, after correction for spectral overlap. Previously, we have used ^23^Na-CSI to study myocardial ischemia and found increases in Na_i_^+^ signal of 200–300 %, where overlap with the Na_e_^+^ signal had negligible effects on Na_i_^+^ signal intensity [23, 28].

With CE-MRI at 60 and 140 mmHg (Fig. 6), the myocardium appeared dark in the T_1_ weighted image prior to Gd perfusion, whereas it appeared brighter after Gd perfusion, confirming the arrival of Gd in the myocardium. In addition, higher signal intensities of the mid-axial slice of the LV myocardium and the hyperenhanced-subendocardial area (Fig. 7) were observed at 140 mmHg compared to perfusion at 60 mmHg. One might assume a similar increase in extracellular Gd content as observed for Na_e_^+^. However, Gd affects both intra- and extracellular water, and therefore the effects of the increased extracellular Gd content are diluted compared to Na_e_^+^. Furthermore, the relationship between [Gd] and signal intensity is complex and non-linear.

We consistently observed transmural heterogeneous enhancement of the LV myocardium (Figs. 6, 7) after perfusion with Gd. The subendocardial area showed higher signal intensity, suggesting more edema in the subendocardial area than in the subepicardial area. This is most likely related to the intramyocardial pressure gradient, with this pressure being higher in the subendocardial area. In the model used, hydrostatic pressure difference is the most important, if not the only, source of edema, whereas in vivo differences in microvascular permeability and osmotic pressure may also contribute to the formation of edema [17]. The subendocardial area with higher signal intensity was significantly larger in hearts perfused at 140 mmHg than at 60 mmHg, confirming more edema at higher perfusion pressure. This finding might provide an explanation for observed late enhancement in patients with arterial hypertension in the absence of myocardial infarction [29]. Interestingly, a similar transmural distribution of signal intensities as observed in the CE-MR images can also be seen in the Na_e_^+^ images (Fig. 5). However, since these hearts belong to different groups and, in addition, differences in spatial resolution, slice thickness and cardiac gating exist between CE-MRI and ^23^Na-CSI groups, direct correlation of these patterns is not possible.

Whether ^23^Na-CSI is indeed better capable than CE-MRI to distinguish between normal, edematous and infarcted tissue, in acute and chronic myocardial infarction, requires further experiments in an infarct model.

### Methodological considerations

- In addition to intracellular and interstitial contributions, the signals from the total water space marker DMMP and the extracellular space marker PPA include also contributions from vasculature, ventricular cavities, and perfusion and suction tubing adjacent to the rat heart. Nevertheless, our results clearly suggest a significant increase of the myocardial extracellular space within an hour of perfusion at higher pressure.
- In biological tissue, ^23^Na (in Na^+^ ions) is present in a much lower concentration than ^1^H in H_2_O. This explains, together with the lower MR sensitivity of ^23^Na, the low spatial and temporal resolution of our ^23^Na-images compared to the ^1^H-MR images. To increase the SNR, we have chosen a thicker slice and bigger voxel size, and we have used k-space weighted acquisition to avoid Gibbs ringing. Moreover, the fact that ^23^Na has short relaxation times enabled us to use short repetition times. To partially overcome the low spatial resolution in the ^23^Na-images, we have used ^1^H-MR images as anatomical reference.
- In this model, myocardial interstitial edema might already be present at 60 mmHg, resulting in a larger extracellular space than observed in vivo. However, the observed increase of Na_e_^+^ at 140 mmHg clearly suggests a further expansion of the extracellular compartment, which was corroborated by ^31^P- and ^1^H-MR data.
- Na_e_^+^ mostly shows mono-exponential T_2_ relaxation (T_2_ = 38.7 ms, Van Emous et al. [24]) whereas Na_i_^+^ shows bi-exponential T_2_ relaxation with usually 60 % of the signal fast relaxing (T_2f_ = 2.3 ms [24]) and 40 % slow relaxing (T_2s_ = 18.9 ms [24]). The CSI method requires an acquisition delay (0.91 ms), which is in the range of the T_2f_ component, causing a non-negligible loss of Na_i_^+^ signal. However, although Van Emous et al. have observed changes in T_2_ relaxation during ischemia and reperfusion, no such changes have been observed during normal perfusion such as used in the current experiments. Therefore, no correction was applied. Similar arguments apply with respect to T_1_ relaxation.
- It is important to mention that our CE-MRI protocol is different from that used clinically to assess infarct size. We have used continuous perfusion of contrast agent, whereas in the clinic a bolus of contrast agent is administered. Due to the absence of an infarct and due to time constraints caused by the high rat heart rate, nulling of ‘remote’ myocardium is not possible in our model.

### Clinical implications

With a non-infarct model of edema, we have demonstrated that tissue edema shows up in CE-MRI. This suggests that CE-MRI might lead to wrongful diagnosis of infarction in other cardiac disease states exhibiting edema or to overestimation of infarct size [1, 2]. ^23^Na-CSI might be an attractive tool to resolve these issues. We have shown that viable—but edematous myocardium—is characterized by increased Na_e_^+^ and normal Na_i_^+^ signals. Previously, we have shown that during global low flow and complete regional ischemia, Na_i_^+^ signals increase several-fold, with partial recovery upon reperfusion [23]. Furthermore, we have previously observed in a model of chronic infarction after coronary artery ligation, a substantial increase of the Na_e_^+^ signal and a complete absence of Na_i_^+^ signal in the infarct area [30, 31]. These characteristics make ^23^Na-CSI a promising tool to discriminate between edema, acute and chronic MI. So far, only total Na^+^-MRI has been used clinically. However, to make ^23^Na-CSI a clinically feasible, high field (7 T) MRI scanners are recommended and a shift reagent safe for human use is required, which is not yet available, but could be developed, since MR contrast agents and MR shift reagents are chemically rather similar. Alternatively, the use of multiple quantum filtered MR methods [32, 33] or ultrashort-echo time (UTE) sequences [34] could be investigated.
